# Web Data Mining: Validity of Data from Google Earth for Food Retail Evaluation

**DOI:** 10.1007/s11524-020-00495-x

**Published:** 2020-11-23

**Authors:** Mariana Carvalho de Menezes, Vanderlei Pascoal de Matos, Maria de Fátima de Pina, Bruna Vieira de Lima Costa, Larissa Loures Mendes, Milene Cristine Pessoa, Paulo Roberto Borges de Souza-Junior, Amélia Augusta de Lima Friche, Waleska Teixeira Caiaffa, Letícia de Oliveira Cardoso

**Affiliations:** 1 National School of Public Health, Fiocruz-RJ, Rua Leopoldo Bulhões, 1480- Manguinhos, Rio de Janeiro, 21041-210 Brazil; 2 Instituto de Comunicação e Informação Científica e Tecnológica em Saúde, Fiocruz-RJ, Av. Brasil, 4.365 - Manguinhos, Rio de Janeiro, 21040-900 Brazil; 3 Department of Nutrition, Universidade Federal de Minas Gerais, Av. Alfredo Balena 190, Belo Horizonte, MG 30130-100 Brazil; 4 Faculdade de Medicina, Universidade Federal de Minas Gerais. Observatório de Saúde Urbana, Av. Alfredo Balena 190, Belo Horizonte, MG 30130-100 Brazil

**Keywords:** Food environment, Food retail, Validation study, Geocoding services, Google Earth, Urban health

## Abstract

To overcome the challenge of obtaining accurate data on community food retail, we developed an innovative tool to automatically capture food retail data from Google Earth (GE). The proposed method is relevant to non-commercial use or scholarly purposes. We aimed to test the validity of web sources data for the assessment of community food retail environment by comparison to ground-truth observations (gold standard). A secondary aim was to test whether validity differs by type of food outlet and socioeconomic status (SES). The study area included a sample of 300 census tracts stratified by SES in two of the largest cities in Brazil, Rio de Janeiro and Belo Horizonte. The GE web service was used to develop a tool for automatic acquisition of food retail data through the generation of a regular grid of points. To test its validity, this data was compared with the ground-truth data. Compared to the 856 outlets identified in 285 census tracts by the ground-truth method, the GE interface identified 731 outlets. In both cities, the GE interface scored moderate to excellent compared to the ground-truth data across all of the validity measures: sensitivity, specificity, positive predictive value, negative predictive value and accuracy (ranging from 66.3 to 100%). The validity did not differ by SES strata. Supermarkets, convenience stores and restaurants yielded better results than other store types. To our knowledge, this research is the first to investigate using GE as a tool to capture community food retail data. Our results suggest that the GE interface could be used to measure the community food environment. Validity was satisfactory for different SES areas and types of outlets.

## Introduction

The food retail environment is a component of the food environment. It includes the density and type of retail food outlets (community environment component) and availability, quality and location of food products within such outlets (consumer environment component). Researchers, health professionals and policymakers are increasingly recognizing the role of the retail food environment in driving health behaviours and outcomes [1].

However, a scarcity of accurate retail environmental data remains a challenge for researchers around the world [2]. The lack of consistency and rigour in measuring food retail environments is believed to contribute to the inconsistent results of studies investigating the diet-food environment relationship [3, 4]. Poor data can lead to uncertainty, bias and reduced statistical power [5]. In addition, when stakeholders have inaccurate information about the food environment, their planning, policies and efforts to improve access to healthy food can be adversely affected [6].

The most common methods of obtaining information on community food outlets are direct field observations (primary data collection) and the use of secondary data [5]. For reasons of pragmatism and efficiency, most studies have relied on secondary data from administrative and commercial databases, telephone and Internet directories and omnidirectional imagery such as Google Street View [2, 3, 5]. However, the use of secondary databases as a source of information on food outlets without validation raises quality concerns and is a significant limitation of food environment studies [3, 5–7].

Primary data sources (i.e. data collected through direct observations made by researchers) is considered the gold standard in characterizing food retail environments [3]. However, such methods have a number of disadvantages: the fieldwork process is costly, is time- and labour-intensive (particularly for studies that cover wide geographic areas) and cannot be used for retrospective analyses [7, 8].

To overcome the challenge of obtaining accurate data on community food environment due to the aforementioned problems with existing primary and secondary data sources, we developed an innovative tool designed to automatically capture points of interest (POIs) in a specified area (such as food retail data) from Google Earth (GE). While existing primary and secondary data sources cannot cover large geographic regions, GE web data is available worldwide in multiple languages, may be captured for large areas and is fairly reliable, even in middle-income settings [9]. Therefore, GE enables the compilation of data sets that could not be otherwise acquired. However, despite the widespread availability of web sources, to our knowledge no other study has yet utilized and validated data from GE to characterize food outlets. Showing these data to be valid would indicate that GE could be a faster, cheaper way of collecting data for studies about community food retail and pave the way for the platform to become an important data source for such studies.

Thus, in order to advance the science on measuring the community food retail environment, we tested the validity of web source data for the assessment of food outlets by comparison to ground-truth (gold standard) data in two Brazilian cities, Rio de Janeiro and Belo Horizonte. As a secondary aim, we tested whether validity differs by type of food outlet and area socioeconomic status (SES), as measured by the Health Vulnerability Index. Our a priori hypotheses were (1) that the GE data is valid (with sensitivity and positive predictive values of at least 0.51); (2) that the validity is better for supermarkets and restaurants compared to small food outlets; and (3) that the validity is worse in areas of high vulnerability for health problems.

## Materials and Methods

### Settings

The study covered Rio de Janeiro and Belo Horizonte, two of the largest cities in Brazil located in the country’s southeast region. Rio de Janeiro municipality, the capital of the eponymous state, is the second most populous city in Brazil. Covering an area of 1224.56 km^2^, Rio de Janeiro has an estimated population (in 2010) of 6,320,446, a population density of 5265/km^2^ and a Human Development Index (HDI) of 0.761. Belo Horizonte is the capital of the state of Minas Gerais and Brazil’s sixth most populous city. It has a population of 2,375,151 living in a 331.401km^2^ area, a population density of 7167/km^2^ and a HDI of 0.810.

### Sampling

For sampling purposes, data from the National Census of 2010 and the National Register of Addresses for Statistical Purposes (CNEFE) were used. For each municipality, the sample range consisted of all census tracts with at least 50 permanent private households or 10 outlets registered with the CNEFE as “Establishments for other purposes”.

The census tracts were classified according to the Health Vulnerability Index (HVI). The HVI is a composite index that covers aspects related to sanitation, housing, education, income and work. It enables the identification of areas with deprived socioeconomic conditions within a given urban space and classifies the census tracts into four strata of vulnerability: stratum 1, low risk (at least 0.5 standard deviation (SD) below HVI mean for each municipality); stratum 2, medium risk (0.5 SD below mean to 0.5 SD above mean); stratum 3, high risk (0.5 SD above mean to 1.5 SD above mean); and stratum 4, very high risk (at least 1.5 SD above mean) [10]. The sample was stratified according to HVI to ensure that at least 12 census tract areas with very high risk were included, since this stratum represents 8% of the all the census tracts but has specific characteristics that are of interest for the purposes of this study. The census tracts were randomly selected, and the number of census tracts selected in each HVI level was proportional to the number of census tracts in each category. The sample size for each municipality was calculated as 150 census tracts (which corresponded to 9.14 km^2^ for Belo Horizonte and 8.38 km^2^ for Rio de Janeiro), considering an average of four food outlets per tract. A sample of 600 food outlets was expected, allowing a sensitivity estimate of around 80% with a sample error of approximately 3%.

### Google Earth Data Acquisition

We developed a tool for the automatic acquisition of community food retail data (or other POIs) through the GE web service. The method consists of the generation of a regular grid of points covering all of the study areas. The distance between the points defines the cell size of the grid—the lower the distance, the higher the resolution. The resolution of the grid is empirically determined and defined by the density of food retail outlets—an area with a high density of outlets requires a high grid resolution. For each point in the grid, we submitted a query via URL with specific search terms for food retailers, e.g. “restaurant”, “bar”, “supermarket”.

**Fig. 1:**
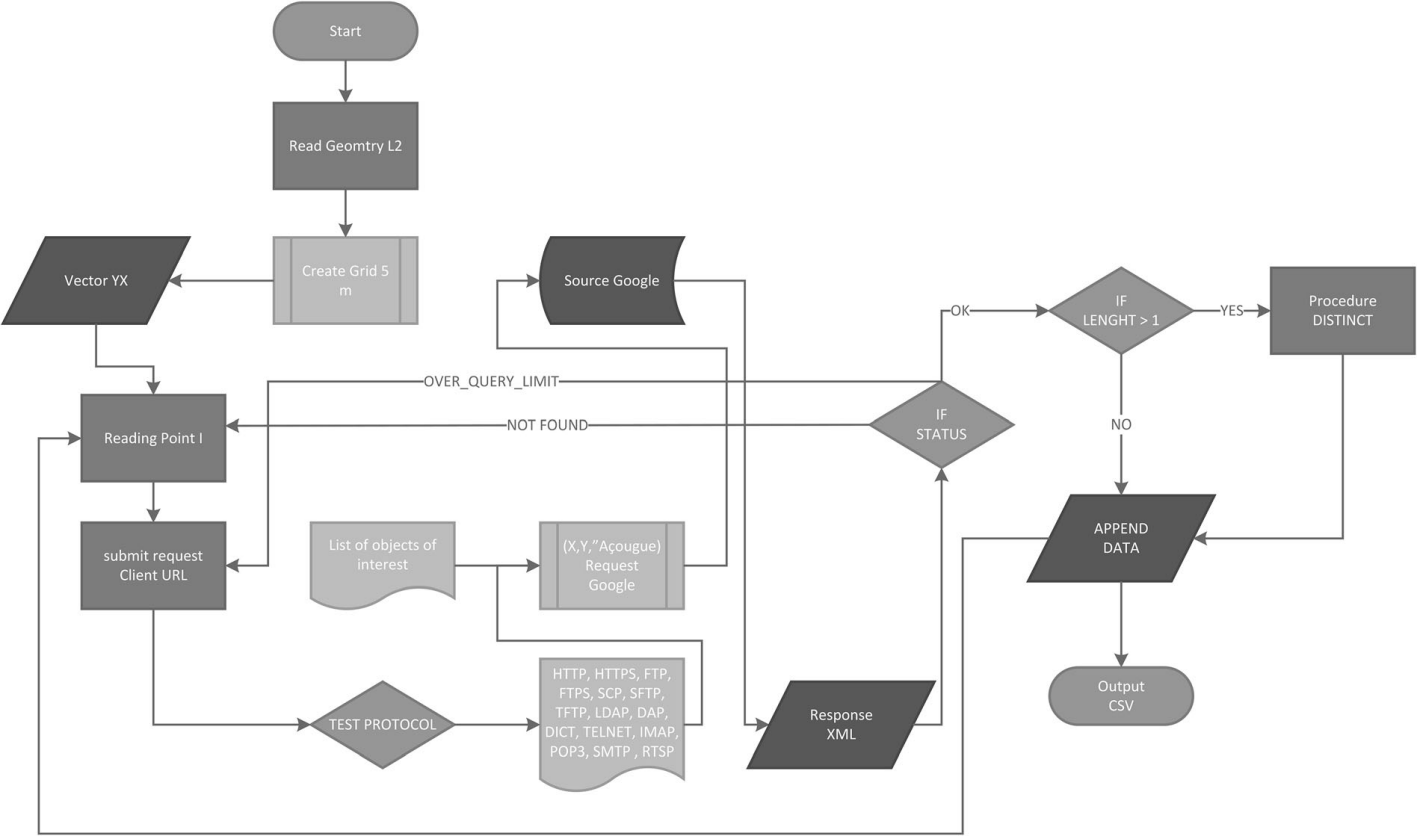
Algorithm for the acquisition of food retail outlets data in Google Earth

If the term is not found for a given point, the algorithm (Fig. 1) jumps to the next point until all the points in the grid have been visited. The algorithm tests for duplication of data, so each food retail outlet is registered only once in the output file. This file, in JSON format, contains the names, addresses, categories in the GE database and geographical coordinates (latitude and longitude) in the WGS84 of all the food retail outlets in the study area.

We used the National Classification of Economic Activities (CNAE) to define the type of food retail outlets to be searched in the GE database. The CNAE is an instrument for the national standardization of economic activity codes and is the basis of the criteria used by the various organs of the Brazilian Tax Administration. We searched all outlets in which the sale of food is the primary activity, including supermarkets, fruit and vegetable markets, bakeries, convenience stores, corner stores, small markets, grocery stores, butchers, fish/seafood markets, live poultry stores, restaurants, bars, liquor stores, fast food outlets, cafeterias, delis, health food stores and confectionary stores. We used a broad search strategy that expanded the original terms described in CNAE; for instance, for small grocery stores, we used the terms “mercearia”, “mini-mercado” and “armazém” (in Portuguese). In order to properly select the terms and use this broad strategy, a qualitative inventory of terminology for food outlet types in Brazil was conducted with different research groups across the country.

We excluded outlets in which selling food was not a primary activity, such as department stores (CNAE 47130) and pharmacies (CNAE 47717). We also excluded food retail outlets inside institutions (e.g. schools and universities) because they are not freely available to the general public and so do not constitute part of the community food environment, but rather the organizational food environment [3, 11].

Our tool was implemented in R version 3.2.4, under fair use, and the proposed method has potential non-commercial or scholarly research applications. The acquisition through the GE web service was obtained in the week prior to data collection in each census tract.

### Gold Standard: Ground-Truth Observations

A ground-truth approach involving primary data collection of food retail outlet types and addresses was used by trained observers not guided in the field by the GE list of food retail outlets that was to be tested [3]. A systematic canvassing of the study area (150 census tracts in each municipality) was conducted. To ensure that all outlets from each area were registered, the team went through all the census tracts according to the Sector Route method as outlined in the Brazilian Institute of Geography and Statistics (IBGE) methodology [12]. The Sector Route enabled observers to walk past every address and store in a given area in a disciplined, careful manner. For example, observers were instructed to interpret the census tract maps. After locating their position on the map and verifying that they were located within the sector boundaries, the observer would then walk a pre-defined route from its starting point within the sector, walking along all the street faces of the selected block while always maintaining the work area beyond their right shoulder. One census tract area was covered at a time and all food stores were recorded. Detailed information about the Sector Route method can be obtained at IBGE, 2010 [12].

Data were collected on foot. The observer did not enter into the outlets, but did list their names and addresses and classified each food outlet type based on characteristics observed from the outside and at the entrance looking in.

The ground-truth data acquisition was conducted between September and November 2018 by a research team of nutritionists and nutrition undergraduates. Quality control procedures included the creation of a surveying protocol and instructional textbook, theoretical and practical staff training, frequent communication between the field team and coordinators and a review of the completed questionnaires. Additionally, a pilot of the ground-truth data acquisition instrument examined one census tract prior to data collection in order to test and adapt the instrument.

Outlets that were closed or had signs indicating that they were under renovation or opening soon were included. We then determined whether these stores were trading, or would be soon, via phone calls or follow-up visits.

### Statistical Analysis

For the ground-truth data, we used the address to geo-reference the location of the food outlet. We had no losses in the geo-referencing process.

For the validity analysis of GE data, we used the ground-truth data as the gold standard for comparison. Following previous studies, the algorithm matched each food retail outlet according to name and geographic location [11]. A food retailer was considered a true positive (TP) if it was listed in both GE and the ground-truth data, a false positive (FP) if it was listed in GE but not in the ground-truth data and a false negative (FN) if it was listed in the ground-truth data but not in the GE. The true negative (TN) refers to those addresses that are correctly classified as a non-food retailer, i.e. addresses negative for food retailers. To calculate TN, we assessed, from the CNEFE, the total addresses (TA) existing in each census tract and excluded those addresses where food retailers were identified in the ground-truth (TP) or in GE (FP) remaining the addresses in the census tracts where neither GE nor ground-truth identified as a food retailer, i.e. TN = TA - TP - FP.

Next, the following measures of validity were calculated:

- Sensitivity: the ratio between true positive/[true positive + false negative]. This represents the probability that the test result is positive, i.e. the proportion of outlets (according to ground-truth data) detected by GE.
- Specificity: the ratio between true negative/[false positive + true negative]. This represents the probability that the test result is negative, i.e. the probability that no outlet is detected where none exists.
- Positive predictive value (PPV): the ratio between true positive /[true positive + false positive]. This represents the probability of an outlet actually existing when the test result is positive, i.e. existing outlets in GE.
- Negative predictive value (NPV): the ratio between true negative/[true negative + false negative]. This represents the probability of an outlet not actually existing when the test result is negative, i.e. outlets that do not exist in GE.
- Accuracy: the ratio between true positive + true negative/[true positive + true negative + false positive + false negative]. This represents the overall probability that the outlets will be correctly classified.

The 95% confidence intervals (CI) for these measures were also calculated. Validity measures were classified as follows: below 0.30 was considered “poor”; 0.31–0.50 “weak”; 0.51–0.70 “moderate”; 0.71–0.90 “good”; and above 0.91 “excellent” [13, 14].

In addition, the validity statistics were compared to the HVI of census tract area (strata 1 (low risk) and 2 (medium risk) vs strata 3 (high risk) and 4 (very high risk)) and to the food outlets category. Based on the context evaluated—i.e. Brazilian cities—we classified the food retail outlets into six categories: (1) supermarkets; (2) convenience stores; (3) restaurants (including bars); (4) natural and fresh food stores (including fruit and vegetable markets, butchers, fish/seafood/chicken markets and health food shops); (5) ultra-processed food stores (including fast food outlets, pizzerias, cafeterias, delis, snack bars, confectionary stores, liquor stores and ice cream parlours); and (6) other small and local markets, e.g. corner stores, mini-markets, and bakeries [15].

Due to the small expected values, the differences between TP vs FN and FP for ground-truth data compared to GE interface data were assessed using Fisher’s exact tests.

Statistical analyses were conducted using Stata, version 14 (StataCorp, College Station, TX, USA) and MedCalc version 19.0.7 (MedCalc Software bvba, Ostend, Belgium).

## Results

**Fig. 2:**
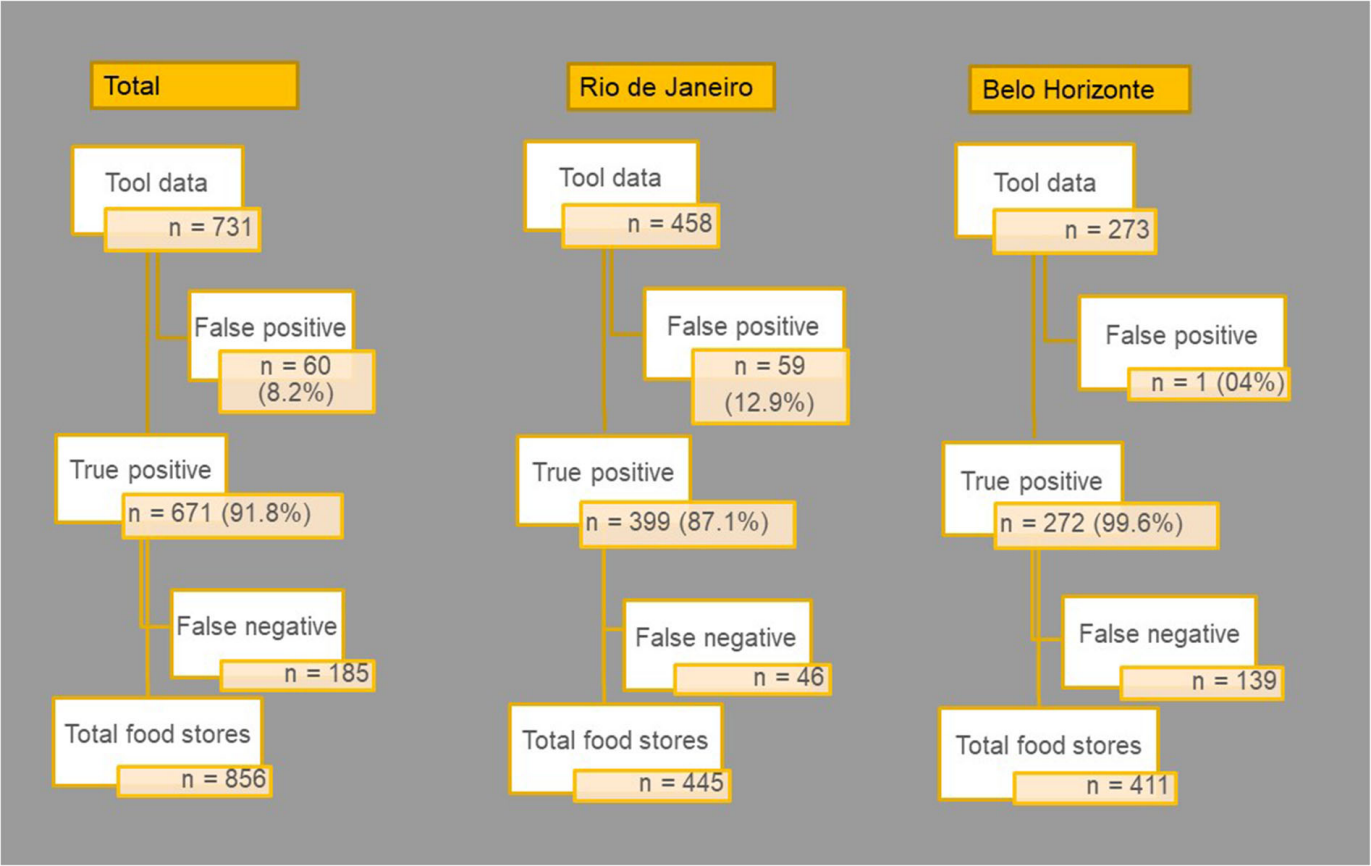
Counts of food outlets during validation process.

Figure 2 reports the number of food outlets for the total sample as well as for each city, showing comparisons between ground-truth and GE sources. Overall, 856 outlets were identified in the ground-truth data (445 in Rio de Janeiro and 411 in Belo Horizonte) in 285 census tracts, compared to 731 outlets in the GE interface data (458 in Rio de Janeiro and 273 in Belo Horizonte). While food outlets were more likely to be missing in Belo Horizonte (false negative = 139), they were overestimated by GE in Rio de Janeiro (false positive = 59) (Fig. 2). Fifteen census tracts were not visited due to safety concerns (7 in Rio de Janeiro and 8 in Belo Horizonte).

In both cities, the GE interface data scored moderate to excellent compared to the ground-truth data across all the validity measures: sensitivity, specificity, PPV, NPV and accuracy (Table 1).

**Table 1:** Validity of the test method (web interface source) compared with the gold standard method (ground-truth)

Validity statistics	Total % (95% CI)	Rio de Janeiro % (95% CI)	Belo Horizonte % (95% CI)
Sensitivity	78.4 (75.5, 81.1)	89.7 (86.5, 92.3)	66.3 (61.5, 70.9)
Specificity	99.8 (99.7, 99.8)	99.3 (99.2, 99.5)	100 (100, 100)
Positive predictive value	91.8 (89.6, 93.7)	87.1 (83.7, 90.0)	100 (98.7, 100)
Negative predictive value	99.3 (99.2, 99.4)	99.5 (99.3, 99.6)	99.1 (99, 99.3)
Accuracy	99.1 (98.9, 99.2)	98.9 (98.7, 99.1)	99.2 (99, 99.3)

**Fig. 3:**
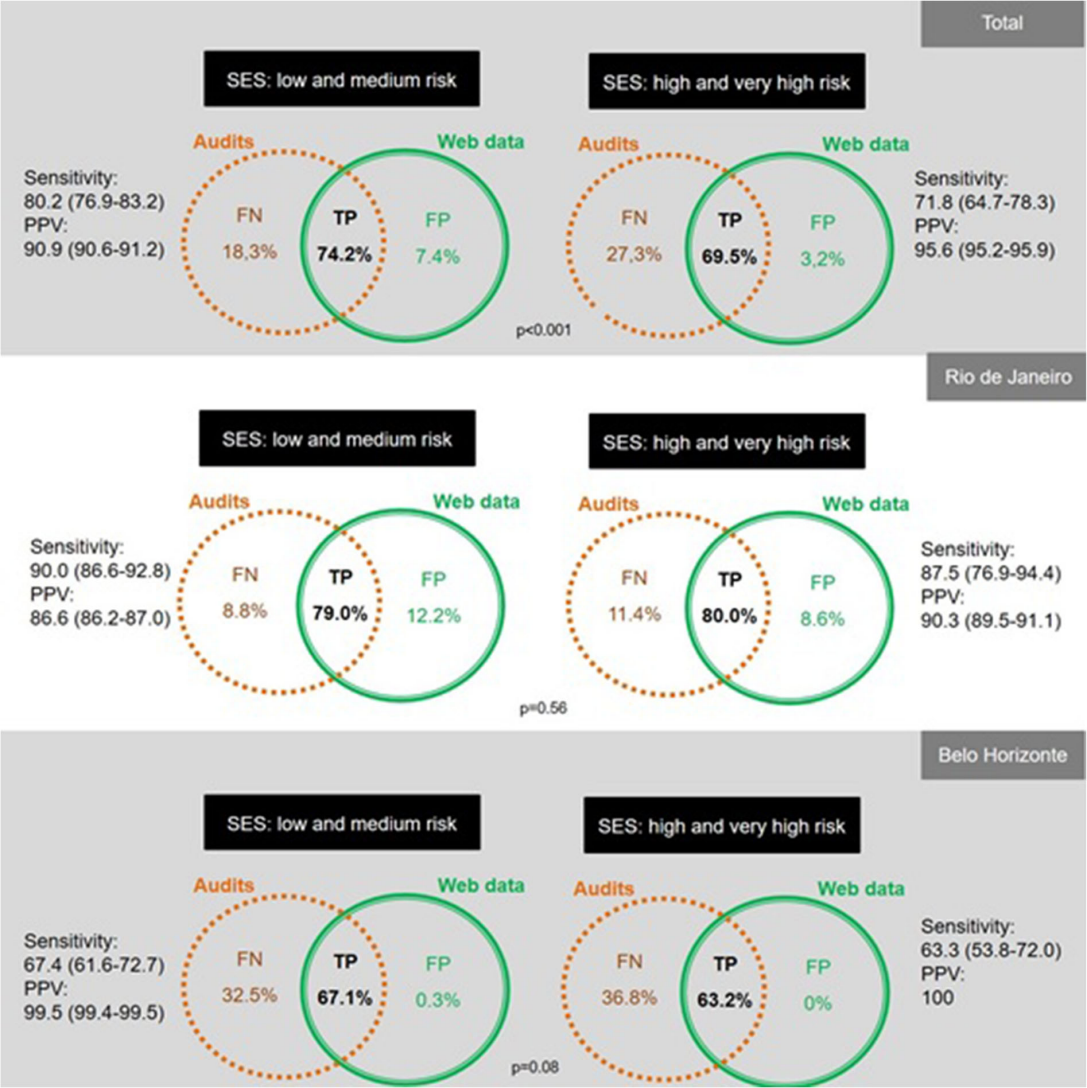
Results of the field validation across socioeconomic conditions (the Health Vulnerability Index). Key: SES = socioeconomic status (measured here through the Health Vulnerability Index); TP = true positive; FN = false negative; FP = false positive; PPV = positive predictive value

Overall, sensitivity was satisfactory (moderate to excellent) and similar across different types of outlets. Supermarkets, convenience stores and restaurants presented TPs at a higher rate. On the other hand, ultra-processed food stores and small and local markets presented a slightly worse sensitivity (Table 2). The census tracts with low and medium HVI vs high and very high HIV presented similar results, with most sensitivity and PPV being classified as good and excellent (Fig. 3). No spatial pattern was identified (data not shown).

**Table 2:** Results of the field validation across food stores type

Validity statistics	Supermarkets	Convenience stores	Restaurants	Natural and fresh food stores	Ultra-processed food stores	Small and local markets
Total						
True positive (*n*) %	(17) 94.4%	(18) 94.7%	(396) 83.4%	(29) 78.4%	(128) 66.7%	(64) 71.1%
False negative (*n*) %	(1) 5.6%	(1) 5.3%	(79) 16.6%	(8) 21.6%	(64) 33.3%	(26) 28.9%
Sensitivity % (95% CI)	94.4 (72.7–99.9)	94.7 (73.9–99.9)	83.4 (79.7–86.6)	78.4 (61.8–90.2)	66.7 (59.5–73.3)	71.1 (60.6–80.2)
Rio de Janeiro						
True positive (*n*) %	(13) 100%	(12) 100%	(256) 89.5%	(5) 100%	(60) 90.9%	(35) 79.5%
False negative (*n*) %	(0) 0.0%	(0) 0.0%	(30) 10.5%	(0) 0.0%	(6) 9.1%	(9) 20.5%
Sensitivity % (95% CI)	100 (75.3–100)	100 (73.5–100)	89.5 (85.4–92.8)	100 (47.8–100)	90.9 (81.3–96.6)	79.6 (64.7–90.2)
Belo Horizonte						
True positive (*n*) %	(4) 80.0%	(6) 85.7%	(140) 74.1%	(24) 75.0%	(68) 54.0%	(29) 63.0%
False negative (*n*) %	(1) 20.0%	(1) 14.3%	(49) 25.9%	(8) 25.0%	(58) 46.0%	(17) 37.0%
Sensitivity % (95% CI)	80.0 (28.4–99.5)	85.7 (42.1–99.6)	74.1 (67.2–80.2)	75.0 (56.6–88.5)	53.9 (44.9–62.9)	63.0 (47.6–76.8)

## Discussion

This study assessed the validity of GE food retail data as compared with the gold standard of ground-truth data in two large Brazilian cities. To our knowledge, this research is the first to investigate GE as a tool for capturing community food retail environment data and adds to the literature by revealing a novel potential option for the objective measurement of the food environment. Overall, the GE data was assessed as being of moderate to excellent validity.

Of late, researchers have started to use freely available data from online geo-referencing services (such as GE, Google Maps and OpenStreetMap (OSM)) to evaluate health-related features of the built environment [9, 16, 17]. Lemke et al. (2015) [16] compared two geo-referencing services (the Google Geocoding Application Programming Interface and OSM) for residential address quality and found that Google’s geo-referencing was superior to that of OSMs. Another study revealed that virtual measures of Google Maps offered high validity and reliability for assessing obesogenic built environments (including pavements, road structures, bus stops and green spaces) [9]. A study conducted in Germany found reasonable validity of Google Maps and OSM data in regard to environmental obesogenic factors related to diabetes surveillance (variables investigated included restaurants, universities, schools, hospitals and cafés). Sensitivity ranged from 33 to 96% but was not presented (or shown) for every category [17]. In addition, a systematic review conducted in 2018 that investigated the use of Google Street View (GSV) in health research revealed that its most common application was in the assessment of the neighbourhood built environment. This review concluded that, despite important limitations, GSV is a promising tool for the automated assessment of environments for health research [18].

Since our study is the only one we are aware of that aims to test GE as a source of capturing community food retail environment data, no comparison studies are available. Much of the existing literature on the community food environment has relied on secondary data, such as commercial lists. In our study, GE data was shown to be least as good as other secondary commercial data sources. A 2017 meta-analysis of the validity of commercial business data on food outlets found a broad range of secondary data quality, although most studies concluded that quality was moderate (0.41–0.60) to substantial (0.61–0.80). The sensitivity and PPV found in the present study were higher than the median reported by this meta-analysis (PPV = 77% and sensitivity = 60%) [19].

Contrary to our initial hypothesis, the present study did not find a clear relationship between social vulnerability and validity of GE. This finding is similar to the results of other studies using secondary data [11, 13, 14]. In the 2017 meta-analysis of the validity of commercial business data, seven of the nine studies that examined neighbourhood socioeconomic status showed that there were no significant differences in validity across neighbourhoods [19]. Since our initial hypothesis that “the validity is worse in areas of high vulnerability for health problems” was refuted, with both areas presenting satisfactory validity measures in our study, these results may be considered encouraging.

However, the present study did find slightly divergent results according to type of food outlets. Supermarkets, convenience stores and restaurants presented better results, while ultra-processed food stores and natural food stores presented worse validity parameters, although this varied by city. This is in line with other studies that found validity statistics for supermarkets, convenience stores and/or restaurants outranked the other types of outlets analysed, with sensitivity ranging from 58 to 95% for supermarkets, 37 to 87% for convenience stores and 45 to 91% for restaurants [2, 3, 13, 14, 20]. It seems reasonable to suggest that, of the various outlet types, large chain stores (e.g. supermarkets and convenience stores) and outlets with high footfall (e.g. restaurants) may be more accurately identified in secondary data and online geo-referencing services in large part because these types of outlets are linked to recognized brands and may tend to be more permanently established than smaller business.

On the other hand, accuracy may be compromised for other types of outlets. Validation conducted in high-income countries found lower sensitivity and PPV for fruit and vegetable markets, butchers and fishmongers [2, 14]. A study conducted in North Carolina that compared retail food outlet data from two commercial databases to field observations found sensitivity ranging from 0 to 50% for fruit and vegetable markets and cafés [2].

In general, when validity is evaluated according to food outlet types, data for small and independent food outlets perform worse than those of big chain stores [2, 5, 14, 20]. This may be because small stores (including ultra-processed food stores and natural food stores) are more likely to be omitted from commercial databases and online geo-referencing services [2, 5, 14].

Since some categories generated datasets from small sample sizes that may have resulted in some misleadingly extreme results, we should be cautious in interpreting data by outlet classification [19]. Nevertheless, the present study confirmed a previous investigation showing that validity was sensitive to food outlet type [14]. We highlight the importance of considering the accuracy of online geo-referencing services for specific types of food outlets, as has been noted elsewhere for secondary data [14].

Considering the two cities analysed, Rio de Janeiro presented more FP, while Belo Horizonte presented more FN. GE tool overestimated food stores in Rio de Janeiro, and we hypothesize this happened because Rio de Janeiro is a touristic place that could have more information on web data. The communities might be more organized in order to register and make the information available on GE in order to increase the visibility due to tourism.

Several notable points should be raised before recommending the use of GE for capturing food retail data. First, GE cannot detect street vendors, a significant source of food sales in Brazil. Second, the quality of GE data depends on the quality of the outlets’ names and terms used in data acquisition. For the present study, a qualitative inventory of terminology for food outlet types in Brazil was conducted using expert knowledge from ten research groups across the country. This step was considered essential to broad data acquisition. Third, GE does not provide accurate data for food outlet type. Therefore, we validated data in regard to location rather than type. For this reason, we did not provide all validity statistics when we stratified results by outlet type.

Another potential limitation of this study is that the ground-truth data collection was not GPS-assisted, due to the associated costs and safety concerns in both cities. Instead, our ground-truth data categorized outlets based on their external appearance only, which may have resulted in the misclassification of some outlets. To mitigate this issue, information embedded in the outlet name, common knowledge of large food outlet franchises and photography were used to check outlet classification. Finally, our study is limited to large cities in Brazil; therefore, our findings require further assessment before they can be generalized to other contexts. We specially suggest to conduct future studies exploring GE interface in small cities, rural locations and cities with different HDIs.

These limitations notwithstanding, given that we used an innovative interface to obtain food retail data and validated it through comparison with the gold standard of primary data approaches (a ground-truth protocol) in a relatively large sample size comprising of 300 census tracts in two different cities, the present study has important implications for future food environment studies. We believe that the 30 working days we spent ground-truthing 150 census tracts in each city and the cost of approximately $3125 (including only the scholarship of observers and material expenses, excluding the institutional salary of researchers) was a worthwhile investment for these findings.

## Conclusions

Our findings showed that the validity of data from the GE online geo-referencing service was moderate to excellent, suggesting that the GE interface could be used to measure the capturing community food retail environment. Overall, validity was satisfactory and similar across different types of outlets, but better for supermarkets, convenience stores and restaurants. Contrary to expectations, GE performed well for areas of both low and high risk of vulnerability.

We believe that this study offers an important methodological contribution to the science of measuring community food retail environments, especially considering the significant role of changes in food retail environments of Latin American countries (i.e. the rise of food chains and decline of small stores) in increasing access to ultra-processed food, and the nutritional transition (i.e. decrease in undernutrition and increase in cases of overweight) in most countries. The proposed method may be used to capture food retail data in similar cities once the terms used to search for outlets are adapted. Hence, as a next step, we suggest that the method be validated in other countries. This method could also be used to identify and analyse other attributes of built environments, potentially helping researchers and decision-makers to track changes and social inequalities in such environments across locations in a cost-effective way.
